# Peak flow meter with a questionnaire and mini-spirometer to help detect asthma and COPD in real-life clinical practice: a cross-sectional study

**DOI:** 10.1038/s41533-017-0036-8

**Published:** 2017-05-09

**Authors:** Yogesh T. Thorat, Sundeep S. Salvi, Rahul R. Kodgule

**Affiliations:** Chest Research Foundation, Marigold Premises, Kalyani Nagar, Pune 411014 India

## Abstract

Peak flow meter with questionnaire and mini-spirometer are considered as alternative tools to spirometry for screening of asthma and chronic obstructive pulmonary disease. However, the accuracy of these tools together, in clinical settings for disease diagnosis, has not been studied. Two hundred consecutive patients with respiratory complaints answered a short symptom questionnaire and performed peak expiratory flow measurements, standard spirometry with Koko spirometer and mini-spirometry (COPD-6). Spirometry was repeated after bronchodilation. Physician made a final diagnosis of asthma, chronic obstructive pulmonary disease and others. One eighty nine patients (78 females) with age 51 ± 17 years with asthma (115), chronic obstructive pulmonary disease (33) and others (41) completed the study. “Breathlessness > 6months” and “cough > 6months” were important symptoms to detect obstructive airways disease. “Asymptomatic period > 2 weeks” had the best sensitivity (Sn) and specificity (Sp) to differentiate asthma and chronic obstructive pulmonary disease. A peak expiratory flow of < 80% predicted was the best cut-off to detect airflow limitation (Sn 90%, Sp 50%). Respiratory symptoms with PEF < 80% predicted, had Sn 84 and Sp 93% to detect OAD. COPD-6 device under-estimated FEV_1_ by 13 mL (95% CI: −212, 185). At a cut-off of 0.75, the FEV_1_/FEV_6_ had the best accuracy (Sn 80%, Sp 86%) to detect airflow limitation. Peak flow meter with few symptom questions can be effectively used in clinical practice for objective detection of asthma and chronic obstructive pulmonary disease, in the absence of good quality spirometry. Mini-spirometers are useful in detection of obstructive airways diseases but FEV_1_ measured is inaccurate.

## Chronic lung diseases: Differentiating conditions in poorly-equipped settings

A simple questionnaire and peak flow meter measurements can help doctors differentiate between asthma and chronic lung disease. In clinical settings where access to specialist equipment and knowledge is limited, it can be challenging for doctors to tell the difference between asthma and chronic obstructive pulmonary disease (COPD). To determine a viable alternative method for differentiating between these diseases, Rahul Kodgule and colleagues at the Chest Research Foundation in Pune, India, trialed a simplified version of two existing symptom questionnaires, combined with peak flow meter measurements. They assessed 189 patients using this method, and found it aided diagnosis with high sensitivity and specificity. Breathlessness, cough and wheeze were the minimal symptoms required for COPD diagnosis, while the length of asymptomatic periods was most helpful in distinguishing asthma from COPD.

## Introduction

Asthma and chronic obstructive pulmonary disease (COPD) present to a clinician in various forms and usually with non-specific symptoms and signs, leading to significant under-diagnosis and mis-diagnosis. Around 70% of asthmatics in the population aged more than 40 years remain undiagnosed and around 30% of patients diagnosed to have asthma do not have asthma.^1–3^ In India, >95% of patients with COPD remain undiagnosed and around 50% of patients diagnosed to have COPD, may not necessarily have COPD.^4^ The most commonly used objective tool to diagnose asthma and COPD is spirometry. However, spirometry is poorly used in India for several reasons including lack of time, cost, lack of availability, and lack of knowledge.^5^

There have been several attempts to develop simpler diagnostic tools with reasonable sensitivity and specificity that can help detect asthma and COPD in the community and in primary care practice.^6–9^ The sensitivity and specificity reported using these tools ranged between 50 and 96% depending on the criteria used for the diagnosis. The questionnaires for asthma have been tested for assessing prevalence of asthma in the community. The COPD diagnostic questionnaire has been tested for diagnosing COPD in primary care practice. However, these questionnaires are relatively large and scoring system is complex. Also, in real-life practice, primary care practitioners are required to diagnose both asthma and COPD and hence, there is a need for a single questionnaire for detection of both asthma and COPD.

The peak flow meter is a simple, easy to use tool that measures peak expiratory flow (PEF) and detects airflow limitation. Compared to spirometry, peak flow measurements are less-time consuming, are not dependent on trained manpower, easy for patients to perform and are less costly. Although, not as reliable as spirometry, a peak flow meter is a recommended alternative for diagnosis of asthma.^10^ There has been a recent interest in the role of peak flow meter for screening of COPD. Jackson et al. conducted an analysis of the data from the third national health and nutrition survey (NHANES III) and defined a PEFR of <80% predicted as abnormal.^11^ Using this definition they found a sensitivity of 91% and specificity of 82% to detect COPD. From the analysis of the data of the Latin American Project for the Investigation of Obstructive Lung Diseases (PLATINO) study and the Burden of Obstructive Lung Disease (BOLD) study, Perez–Padilla et al. suggested that a pre-bronchodilator PEF of less than 70% of predicted can rule out stages III and IV of COPD.^12^ The reliability of PEF to detect airflow limitation^13^ and COPD^14, 15^ in a community setting has previously been studied. However, the accuracy of peak flow meter to detect both asthma and COPD, in a clinical setting, has not been studied.

Another simpler alternative to spirometry could be the use of handheld mini-spirometers that measure forced expiratory volume in one second (FEV_1_) and forced expiratory volume in six seconds (FEV_6_). Airflow limitation, using mini spirometers, is diagnosed based on the ratio of FEV_1_ to FEV_6_. However, the quality assurance checks for the measurements made by peak flow meter and mini-spirometer are less stringent than those for a standard spirometer and hence, it is important to study their performance compared to standard spirometry. FEV_1_/FEV_6_ measured by standard spirometer has been shown to be a reliable alternative to FEV_1_/FVC for detecting airflow limitation.^16–23^ The reliability of mini-spirometer has been evaluated in at least 3 studies.^24–26^ FEV_1_ measured is used to assess bronchodilator reversibility and classify severity of the disease. Hence, it is important to study the accuracy of a mini-spirometer to measure FEV_1_ in real-life practice.

Previous work on accuracy of peak flow meter and mini-spirometer was carried out separately for asthma and COPD and was population based. In real-life practice, a diagnostic tool should be able to detect both asthma and COPD. Hence, we aimed to study, in real-life practice the sensitivity and specificity of: (i) peak flow meter with and without a questionnaire to detect airflow limitation detected by conventional spirometry and physician diagnosis of OAD, (ii) peak flow meter with and without a questionnaire to predict clinical diagnosis of asthma and COPD and (iii) mini spirometer to detect airflow limitation detected by conventional spirometry.

## Results

**Fig. 1:**
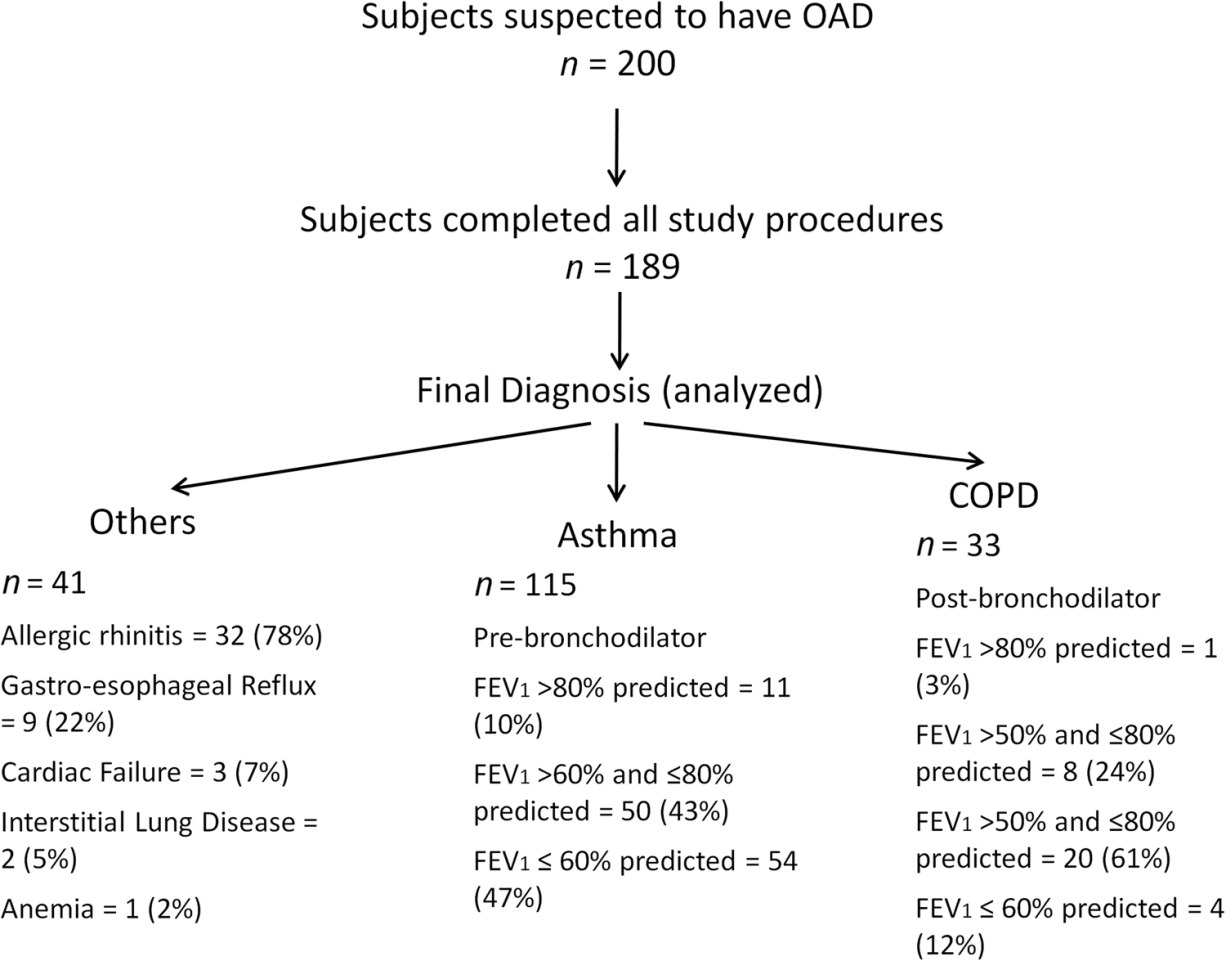
Patient disposition

Two hundred subjects provided consent for study participation out of which 189 completed all the study procedures and were analyzed (Fig. 1). The characteristics of patient population are summarized in Table 1.

**Table 1:** Patient characteristics

	Total	Asthma	COPD	Others
*N*	189	115	33	41
Females, *n*(%)	78 (41.3%)	63 (54.8%)	0 (0%)	15 (36.6%)
Age (years), mean ± SD	51 ± 17	47 ± 16	66 ± 8	50 ± 17
Smoking status				
Non-smoker, *n*(%)	140 (74.1%)	105 (91.1%)	0 (0%)	35 (85.4%)
Current smoker, *n*(%)	14 (7.4%)	3 (2.6%)	10 (30.3%)	1 (2.4%)
Ex-smoker, *n*(%)	35 (18.5%)	7 (6.1%)	23 (69.7%)	5 (12.2%)
Pre-BD FEV_1_, % predicted ± SD	63.1 ± 22.9	62.0 ± 15.6	36.7 ± 12.8	87.3 ± 21.5
Pre-BD FVC, % predicted ± SD	83.6 ± 23.6	81.5 ± 18.1	70.0 ± 15.7	100.2 ± 32.2
Pre-BD FEV_1_/FVC, % ± SD	62.9 ± 18.8	63.2 ± 18.5	43.1 ± 12.0	77.6 ± 05.8

Final physician diagnosis was asthma in 115 subjects, COPD in 33 subjects and “others” in 41 subjects. The diagnosis of patients categorized as “others” included allergic rhinitis (78%), gastro-esophageal reflux (22%), cardiac failure (7%), interstitial lung disease (5%), and anemia (2%).

Table 2 shows the accuracy of each symptom independently for detecting OAD and for detecting asthma and COPD. After stepwise backward logistic regression, “age at cough onset” (*p* = 0.025), “history of wheeze” (*p* = 0.003), and “cough with expectoration” (*p* = 0.031) remained in the final model as significant predictors of diagnosis of OAD. As decided a priori, we retained “breathlessness >6 months” and “cough >6 months” in the model. After addition of these symptoms, only “wheeze” (*p* < 0.01) remained a significant predictor in addition. “Breathlessness >6 months” was the most sensitive (sensitivity = 95%) and “history of wheeze” was the most specific (specificity = 93%) symptoms for the physician diagnosed OAD.

**Table 2:** Accuracy of each symptom (A) for detecting OAD and (B) for detecting asthma in patients who have OAD

Symptom	Sensitivity, %	Specificity, %	Positive predictive value, %	Negative predictive value, %
A Accuracy for detecting OAD				
Breathlessness (mMRC grade ≥ 1)	97.30	75.61	93.51	88.57
Breathlessness > 6 months	94.59	78.05	93.96	80.00
Cough	93.24	78.05	93.88	76.19
Cough > 6 months	89.86	82.93	95.00	69.39
Cough with expectoration	42.57	85.37	91.30	29.17
Wheeze	83.11	92.68	97.62	60.32
B Accuracy for detecting asthma in patients who have OAD				
Wheeze	89.57	39.39	83.74	52.00
Intermittent asymptomatic period	92.17	87.88	96.36	76.32
Smoker	85.22	75.76	92.45	59.52
Pack years < 10 years	90.43	75.76	92.86	69.44
Family history of atopy	53.91	78.79	89.86	32.91
Current age < 40 years	28.70	90.91	91.67	26.79
Age of onset of breathlessness < 40 years	66.09	87.88	95.00	42.65
Age of onset of cough < 40 years	68.70	87.88	95.18	44.62

For differentiating between asthma and COPD only “presence of asymptomatic (no breathlessness, cough, and wheeze) period for >2 weeks” (*p* < 0.01) remained a significant predictor of asthma diagnosis with a sensitivity of 92% and specificity of 88%.

### Peak flow meter with symptoms

**Fig. 2:**
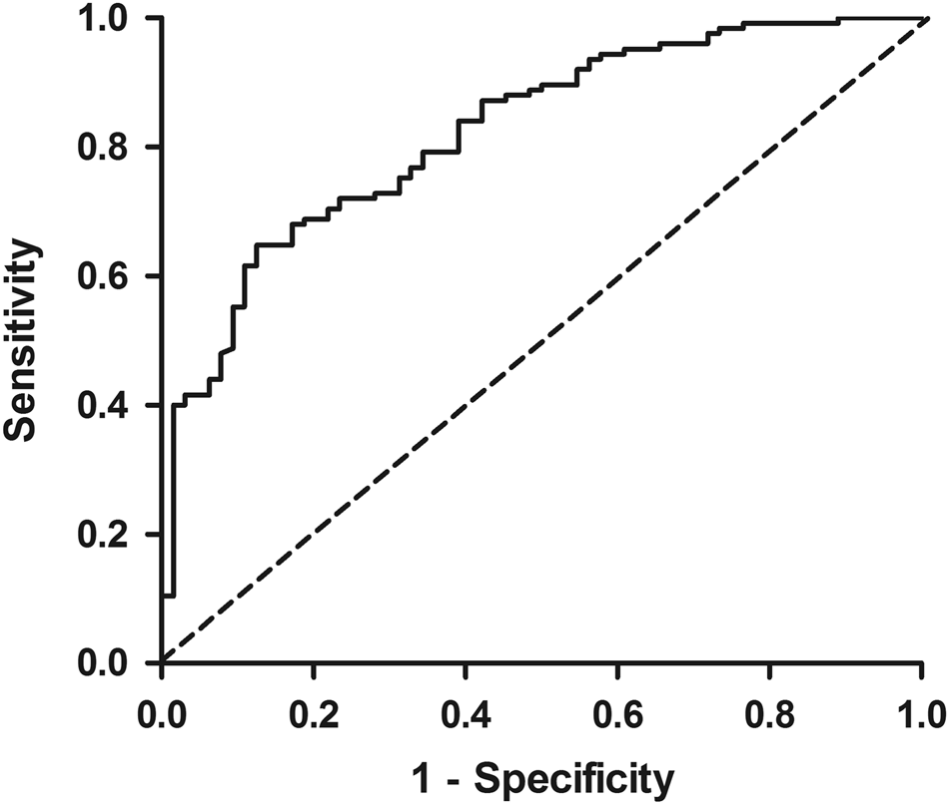
ROC of PEF for detection of OAD

The best cut-off of PEF for detection of spirometry defined airflow limitation (pre-BD FEV_1_/FVC < 0.70) was at ≤80% predicted (Fig. 2), with the AUC 0.82, sensitivity 90%, and specificity 50%.

Similarly, the best cut-off of PEF, for clinical diagnosis of an obstructive airways disease (OAD) was at 80% predicted with AUC 0.85, sensitivity 89%, and specificity 68%. Patients who were detected to have OAD were categorized as asthma if they had “an asymptomatic period >2 weeks in last 1 year” and others were categorized as COPD. Table 3 shows the accuracy of peak flow meter with chronic respiratory symptoms (breathlessness and/or cough >6 months and PEF < 80% predicted) for detection of clinical diagnosis of OAD, asthma, and COPD. Addition of other symptoms did not improve the accuracy over just these two symptoms.

**Table 3:** Accuracy of peak flow meter with and without symptoms for detection of OAD and detection of asthma and COPD

	Sensitivity, %	Specificity, %	Positive Predictive Value, %	Negative Predictive Value, %
Peak flow meter (PEF < 80% predicted) for detection of OAD	89	68	91	62
Peak flow meter and symptoms for detection of OAD (breathlessness and/or cough > 6 months)	84	93	98	62
Peak flow meter and symptoms for detection of asthma (breathlessness and/or cough > 6 months + presence of asymptomatic period)	77	93	95	73
Peak flow meter and symptoms for detection of COPD (breathlessness and/or cough > 6 months + absence of asymptomatic period)	79	95	76	95
2-step model	97	82	95	90

Ninety three percent of patients who did not have OAD were also found to be negative using peak flow meter (i.e., PEF ≥ 80% predicted) with symptoms and 98% of patients detected to have OAD using peak flow meter (PFM) with symptoms actually had an OAD. Similarly, 93% of patients who did not have asthma were also found to be negative using PFM + symptoms and 95% of patients detected to have asthma using PFM + symptoms actually had asthma. 95% of patients who did not have COPD were also found to be negative using PFM + symptoms and 76% of patients detected to have COPD using PFM + symptoms actually had COPD.

Out of 94 patients detected to have asthma using peak flow meter with symptoms, 1 patient (1%) was healthy and 4 (4%) had COPD. Similarly, out of 34 patients detected to have COPD using these tools, 2 patients (6%) were healthy and 6 patients (18%) had asthma.

Since, wheeze had the highest specificity of 93% to detect OAD and breathlessness had highest sensitivity of 95%, we built a 2-step model. In first step, all the patients with wheeze were categorized as OAD. In the second step, all those without wheeze, were categorized as OAD if they had either breathlessness or cough >6 months and also had PEF < 80% predicted. Rest of the patients were categorizes as “No OAD”. This model had 97% sensitivity and 82% specificity to detect OAD (Table 3).

### COPD-6

**Fig. 3:**
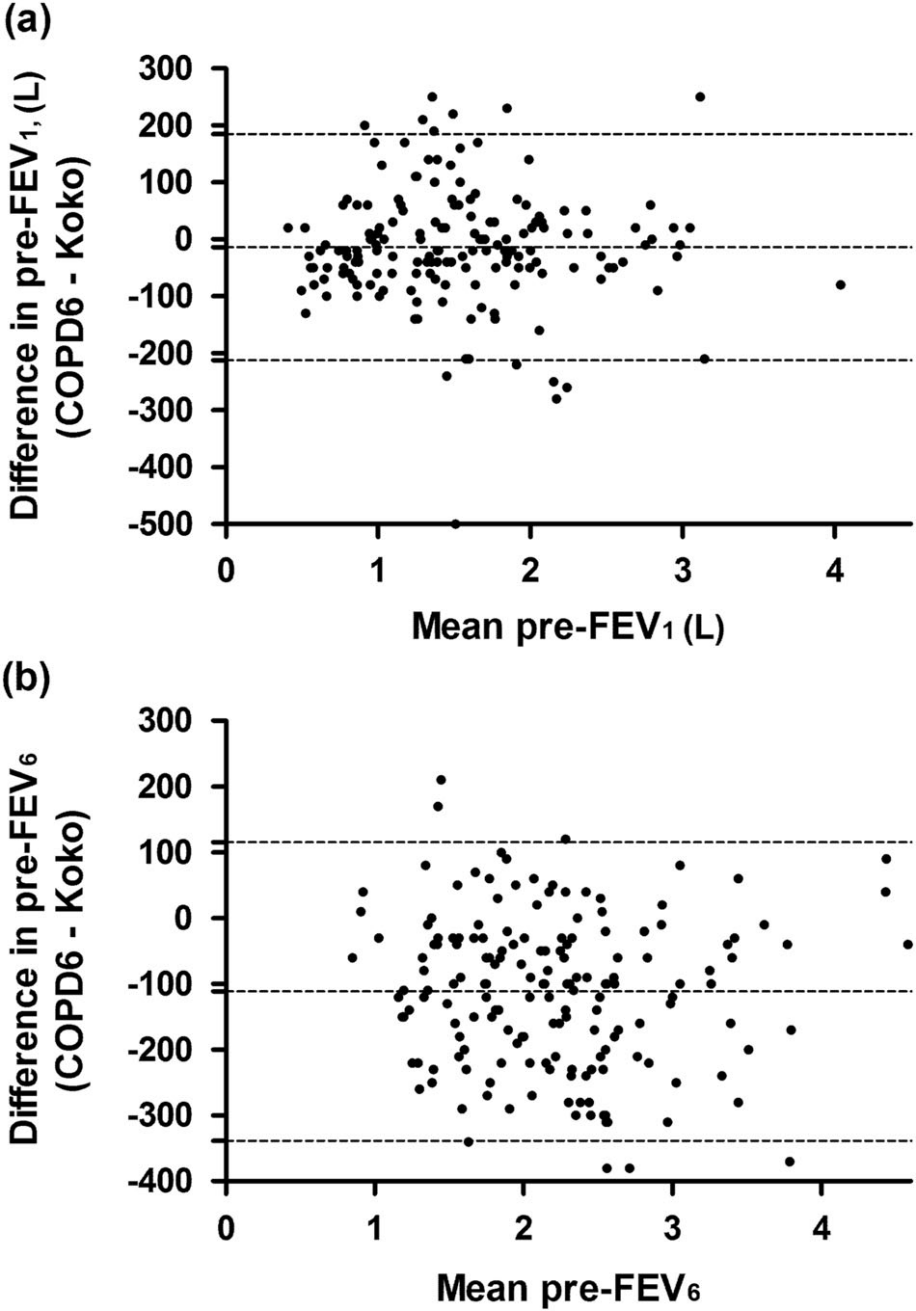
Bland–Altman plot of differences in (**a**) FEV_1_ and (**b**) FEV_6_ measured by COPD6 device and Koko spirometer

On an average, compared to the standard spirometer, the COPD-6 device under-estimated FEV_1_ by 13 mL (95% CI: −212, 185) and FEV_6_ by 112 mL (95% CI: −339, 115) (Fig. 3). The difference in the FEV_1_ and FEV_6_ measured by the two devices was more than 200 mL in 14 (7.4%) and 43 (22.7%) subjects respectively.

**Fig. 4:**
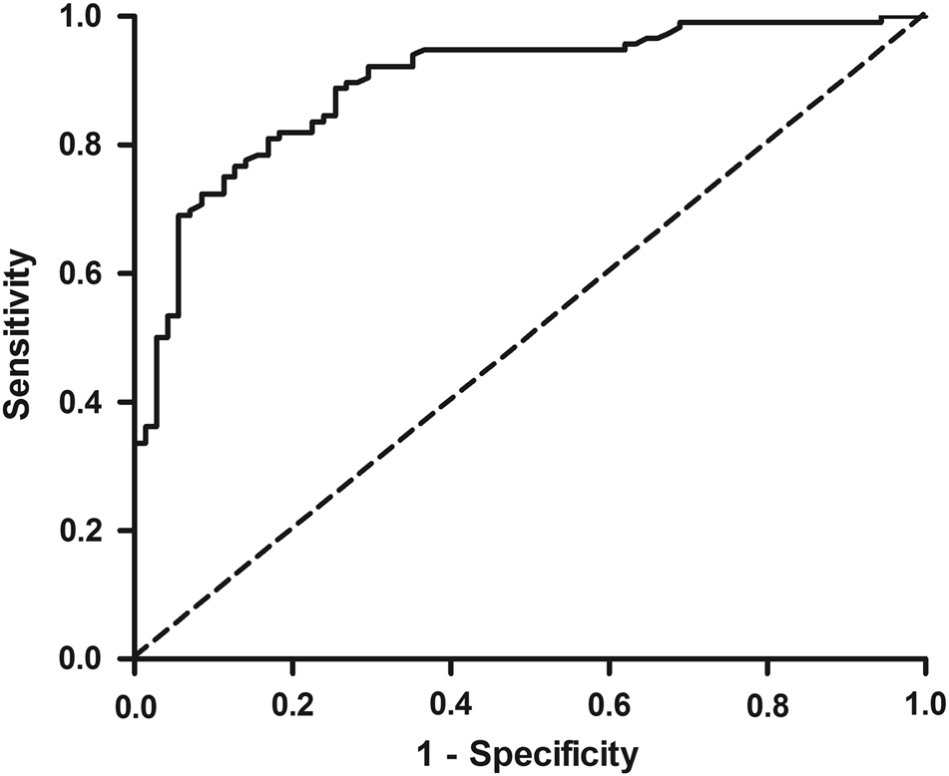
ROC of FEV_1_/ FEV_6_ measured by COPD6 device for detection of OAD

Figure 4 demonstrates the receiver operating curve (ROC) of FEV_1_/FEV_6_ ratio for detection of OAD. At a cut-off of 0.75, the FEV_1_/FEV_6_ had the best accuracy with the area under the curve 87%, sensitivity 80%, and specificity 86%. The sensitivity and specificity at cut-off of 0.70 were 65 and 55%, respectively, and at a cut-off of 0.80 were 88 and 69%, respectively.

In 150 patients (79%), the interpretation of airflow limitation was same by both COPD-6 device and Koko spirometer. 28 (15%) patients had airflow limitation detected by Koko which was not detected by COPD-6 device. Airflow limitation was detected by COPD-6 and not by Koko in 9 patients (5%).

## Discussion

To the best of our knowledge, this is the first study that has assessed the accuracy of a peak flow meter together with a questionnaire and mini-spirometer, to help detect airflow limitation and help diagnose asthma and COPD in real-life practice. Peak flow meter with a cut-off value of PEF < 80% predicted had good sensitivity but low specificity to detect airflow limitation. However, a combination of peak flow meter and a symptom questionnaire increased the sensitivity to 84% and specificity to 93%. This high degree of sensitivity and specificity is encouraging and suggests that a peak flow meter with a questionnaire can be used to detect cases of OAD in clinical practice. Although the use of spirometry will provide the best results, its availability in developing countries is poor and quality of testing done is questionable. Peak flow meter and questionaire being inexpensive, handy, easy to use and less-time consuming are likely to receive larger acceptance than spirometry and may have significant effect in improving diagnosis of OADs.

Use of a peak flow meter and a questionnaire had lower overall sensitivity but good overall specificity for a specific diagnosis of asthma and COPD. Jithoo et al. conducted an analysis of data of 9000 participants from the BOLD study.^27^ The authors reported a sensitivity of 83–84% of PEF to detect COPD, which is comparable to a sensitivity of 89% found in our study to detect OAD. In a prospective cross-sectional survey among 525 participants, Mahboub et al. reported a sensitivity of 73% and specificity of 80% to detect COPD.^15^ However, previous studies were aimed at screening for COPD and were population based. Our study was clinical practice based and was aimed at assessing accuracy of simple diagnostic tools to detect asthma and COPD in patients visiting clinical facility with respiratory symptoms.

In our study we used a short symptom questionnaire for assessing symptoms and for differentiating between asthma and COPD. Presence of breathlessness or cough for more than 6 months along with reduced PEF (<80% predicted) had a reasonably good accuracy to detect patients with OAD. “Presence of asymptomatic period of more than 2 weeks” was the strongest symptom to differentiate between asthma and COPD. Using these questions and PEF measurements, sensitivities, and specificities for reaching a diagnosis of asthma and COPD in patients with respiratory symptoms were around 75 and 95%, respectively. This suggests that these tools can be used effectively, in clinical practice to aid diagnosis of asthma and COPD, in absence of spirometry. Questionnaires have earlier been developed separately for detection of asthma or COPD. However, these questionnaires are bigger and more complex and aimed at detecting these diseases and their risk factors in community settings.^6–9^ We identified minimal symptoms (wheeze, breathlessness/cough, and intermittent relief periods) that were sufficient to provide enough accuracy for disease detection. Being short and simple, these questions would be easy to use in busy clinical practice or by trained healthcare workers. However, being not so elaborate, the information from these tools can play only a valuable supportive role to physician diagnosis.

We also developed a 2-step model for detection of OADs with impressive accuracy measures. However, this model needs to be tested objectively in larger independent studies.

In our study, we found that the mini-spirometer significantly underestimates FEV_1_ and FEV_6_ measured by full-standard spirometry. Gochicoa–Rangel et al. reported a relatively higher (40 mL) mean difference in FEV_1_ measurement and lower (7 mL) mean difference in the measurement of FEV_6_ with much wider confidence intervals.^28^ We found relatively narrow confidence intervals probably because of larger sample size (82 vs. 189) compared to Gochicoa–Rangel’s study. Since, FEV_1_ measured is often used to classify severity of the disease and to assess bronchodilator reversibility, its accurate measurement is important. Although, the mean difference in FEV_1_ measured by the two devices was small, in a significant proportion of patients (7.4%) the difference was more than 200 mL.

Also, the sensitivity (80%) and specificity (86%) to detect a clinical diagnosis of OAD were lower in our study compared to those previously reported. These differences could be because in most of the previous studies FEV_6_ was derived from the same maneuver and using a standard spirometer.^18, 22, 23, 29–31^ Comparatively the sensitivities and specificities of FEV_1_/ FEV_6_ were lower when a mini-spirometer was used.^25, 26^ Since, these mini-spirometer devices do not show flow-volume and volume-time graphs for quality assurance, it is possible that, as a result FEV_1_ and FEV_6_ are often inaccurately measured leading to lower accuracy in detection of OADs.

Eleven patients in our study could not perform standard spirometry, but all of them answered the questionnaire and performed peak flow meter test. Two out of these eleven patients were able to perform mini-spirometry but not standard spirometry. All the 189 patients performed all the study procedures. This suggests wider usability of these tools than standard spirometry.

Our study had some strengths and weaknesses. For the first time we studied the accuracy of peak flow meter with questionnaire and mini-spirometer for detection of asthma and COPD, in one single study. Also, for the first time we studied the accuracy in a clinical setting. The diagnosis of asthma and COPD in our study was based on physician opinion along with evidence of obstruction on spirometry. In few cases additional testing in the form of chest X-ray, body plethysmography, bronchial challenge testing, and DLCO was performed to confirm the diagnosis or rule-out alternative diagnoses.

However, our study was single center based with a smaller sample of 200 patients. Large multi-centric studies are required to replicate our findings. Secondly, our center caters mainly to the patients with asthma and COPD. Hence, a large of patients in this study had OAD. Although, this could have led to sampling bias, having such a facility, allowed to screen adequate number of patients with asthma and COPD. Our findings need to be studied in centers receiving large number of patients with cardiovascular disease and other chronic lung diseases.

We found that only question (“intermittent asymptomatic periods >2 weeks”) was sufficient to differentiate between asthma and COPD, although smoking history and onset of symptoms after the age of 40 years were other important symptoms. Larger studies are required to validate this finding. Besides, we developed tools based on the data from this study and tested their accuracies in the same data. This is likely to provide good internal validity but poor external validity. Hence, these tools need to be tested in independent larger studies.

Also, many patients with COPD do not have any symptom and are hence, likely to be missed using these tools. However, in real-life clinical practice, where reporting patient has some level of symptoms, these tools are likely to be most effective.

## Conclusion

Peak flow meter with few symptom questions can be effectively used in real-life clinical practice for objective detection of asthma and COPD, in absence of good quality spirometry. Mini-spirometers are useful in detection of OADs but FEV_1_ measured is inaccurate. These tools are required to be tested in larger multi-centric studies.

## Methods

In this cross-sectional study we enrolled 200 consecutive adult patients attending the clinical facility of Chest Research Foundation, Pune, with respiratory complains that required spirometry for diagnosis. Patients with history of pulmonary tuberculosis, and those with contra-indications for spirometry, and also pregnant and nursing mothers were excluded from the study. Selected patients were administered a questionnaire developed by the Chest Research Foundation. The questionnaire consisted of seven questions aimed at suspecting OAD and differentiating between asthma and COPD. The questions were history of breathlessness with modified Medical Research Council grade ≥1, cough and wheeze, duration of symptoms, age at the onset of symptoms, whether patient felt complete relief from breathlessness, cough, and wheeze for more than 2 weeks anytime in last 1 year, age, and smoking history.

After questionnaire administration, PEF was measured using a hollow cylinder, EU scale peak flow meter (Cipla Breathometer^®^). Peak flow measurements consisted of at least three acceptable blows after complete inhalation, with the highest two PEF readings repeatable within 40 L/min. The highest PEF was captured as the PEF for the patient. Published reference equations were use to obtain PEF% predicted.^32^

After PEF measurement spirometry was performed using both, the mini-spirometer (Vitalograph^®^ COPD-6 device) and conventional pneumotach spirometer (Koko^®^ Sx) in a randomized sequence. Mini-spirometry was performed according to the instructions in the manufacturer’s manual for COPD-6. Each subject provided at least three acceptable blows with repeatability of 150 mL between highest two FEV_1_ and FEV_6_. The device was checked for calibration at the beginning and at the end of the study. The technician was trained on using the COPD-6 device at the beginning of the study.

Standard spirometry was performed according to the standards published by the American Thoracic Society and European Respiratory Society.^33^ The Koko spirometer was checked for calibration (volume and flow linearity) using 3 L syringe on every day of patient visit. Each subject provided at least three acceptable blows with repeatability of 150 mL between highest two FEV_1_ and FVC. FEV_1_/FVC cut-off of 0.70 was used to define airflow limitation. PEF measurement, COPD-6 and standard spirometry were repeated 15 min after administration of salbutamol 400 mcg using pMDI and spacer. All the tests were performed by the same trained and experienced technician throughout the study.

After reviewing clinical history, physical examination and spirometry reports the physician with a final clinical diagnosis of asthma, COPD, and others. All asthma patients were required to have pre-bronchodilator FEV_1_/FVC < 0.70. Those with normal FEV_1_/FVC ratio and suspected asthma, underwent methacholine challenge test for confirmation of asthma diagnosis. Provocative concentration (PC20) was required to be <8 mg/ml for diagnosis of asthma.^34^ All the patients with diagnosis of COPD were required to have post-bronchodilator FEV_1_/FVC < 0.70 and history of significant exposure to cigarette smoke (>10 pack years) or biomass smoke (>15 years).^35^ In patients with suspicion of asthma and post-bronchodilator FEV_1_/FVC < 0.70, final diagnosis was made based on the values of TLCO. Diagnosis of COPD was made if TLCO was reduced (<lower limit of normal); otherwise, a diagnosis of asthma was made. If required chest X-ray was performed to reach to a final (alternative) clinical diagnosis.^36^

The study was approved by the Institutional Ethics Committee of the Chest Research Foundation, Pune and all the participants provided written informed consent.

### Statistical Analysis

Statistical analysis was performed using IBM’S SPSS Version 20. Assuming a sensitivity and specificity of the diagnostic tool at 75% with a maximum marginal error of estimate of 10%, and with a prevalence of OADs in patient population as per our previous experience as 60%, we estimated that 180 patients will be required.^37^ Considering incomplete data collection of 10% we decided to recruit 200 consecutive patients.

Using stepwise backward logistic regression, we identified the questions for detection of OAD and for differentiation between asthma and COPD. A priori we decided to retain breathlessness and cough in the model, since these questions are important for detection of OADs. A *p*-value of <0.05 was considered as statistically significant.

Using ROC, we obtained best cut-off of PEF and FEV_1_/FEV_6_% predicted for predicting airflow limitation considering FEV_1_/FVC < 0.70 and FEV_1_/FVC less than lower limit of normal on spirometry as standard. Similarly, we obtained best cut-offs of PEF and FEV_1_/FEV_6_ for detection of physician diagnosed OAD.

We then categorized patients as OAD or others, using PEF. Within the subgroup classified as OAD, we categorized the patients as asthma or COPD using questionnaire. We then categorized the entire data as “asthma” and “No asthma” and similarly, “COPD” and “No COPD”. We then obtained the accuracy of PEF with questionnaire to detect asthma and COPD and reported it as overall sensitivities, specificities, PPVs, and NPVs.

We assessed the accuracy of COPD-6 device to measure FEV_1_ and FEV_6_ by comparing the FEV_1_ and FEV_6_ measured by COPD-6 and Koko spirometer using Bland–Altman plot and method.

## References

[1] Gonzalez-Garcia M, Caballero A, Jaramillo C, Maldonado D, Torres-Duque CA (2015). Prevalence, risk factors and underdiagnosis of asthma and wheezing in adults 40 years and older: a population-based study. J. Asthma.

[2] Pakhale S, Sumner A, Coyle D, Vandemheen K, Aaron S (2011). (Correcting) misdiagnoses of asthma: a cost effectiveness analysis. BMC Pulm. Med..

[3] Aaron SD, Vandemheen KL, Boulet LP, McIvor RA, FitzGerald JM, Hernandez P (2008). Overdiagnosis of asthma in obese and nonobese adults. Can. Med. Ass. J..

[4] Lamprecht B, Soriano JB, Studnicka M, Kaiser B, Vanfleteren LE, Gnatiuc L (2015). Determinants of underdiagnosis of COPD in national and international surveys. Chest J..

[5] Vanjare N, Chhowala S, Madas S, Kodgule R, Gogtay J, Salvi S (2016). Use of spirometry among chest physicians and primary care physicians in India. NPJ Prim. Care Respir. Med..

[6] Hansen TE, Evjenth B, Holt J (2015). Validation of a questionnaire against clinical assessment in the diagnosis of asthma in school children. J. Asthma.

[7] Coordination Committee for the International Study of Asthma and Allergies in Childhood (ISAAC). (1992). Manual for the International Study for Asthma and Allergies in Childhood (ISAAC). http://isaac.auckland.ac.nz/phases/phaseone/phaseonemanual.pdf

[8] Stanley AJ, Hasan I, Crockett AJ, van Schayck OCP, Zwar NA (2014). COPD Diagnostic Questionnaire (CDQ) for selecting at-risk patients for spirometry: a cross-sectional study in Australian general practice. NPJ Prim. Care Respir. Med..

[9] Price DB, Tinkelman DG, Nordyke RJ, Isonaka S, Halbert RJ (2006). Scoring system and clinical application of COPD diagnostic questionnaires. Chest.

[10] Global Initiative for Asthma. (2015) Global strategy for asthma management and prevention. p. 149. http://www.ginasthma.org

[11] Jackson H, Hubbard R (2003). Detecting chronic obstructive pulmonary disease using peak flow rate: cross sectional survey. Br. Med. J.

[12] Perez-Padilla R, Vollmer W, Vazquez-Garcia J, Enright P, Menezes A, Buist A (2009). Can a normal peak expiratory flow exclude severe chronic obstructive pulmonary disease?. Int. J. Tuberc. Lung Dis.

[13] Tian J., Zhou Y., Cui J., Wang D., Wang X., Hu G. et al. Peak expiratory flow as a screening tool to detect airflow obstruction in a primary health care setting. *Int. J. Tuberc. Lung Dis* **16**, 674–680 (2012). 10.5588/ijtld.11.0429 22409956

[14] Nelson S. B., LaVange L. M., Nie Y., Walsh J. W., Enright P. L., Martinez F. J. et al. Questionnaires and pocket spirometers provide an alternative approach for COPD screening in the general population. *Chest* **142**, 358–366 (2012). 10.1378/chest.11-1474 22194590

[15] Mahboub B, Alzaabi A, Soriano JB, Salameh L, Mutairi YaL, Yusufali Aa (2014). Case-finding of chronic obstructive pulmonary disease with questionnaire, peak flow measurements and spirometry: a cross-sectional study. BMC Res. Notes.

[16] Akpinar-Elci M, Fedan KB, Enright PL (2006). FEV6 as a surrogate for FVC in detecting airways obstruction and restriction in the workplace. Eur. Respir. J..

[17] Bellia V, Sorino C, Catalano F, Augugliaro G, Scichilone N, Pistelli R (2008). Validation of FEV6 in the elderly: correlates of performance and repeatability. Thorax.

[18] Bhatt SP, Kim Y-I, Wells JM, Bailey WC, Ramsdell JW, Foreman MG (2014). FEV(1)/FEV(6) to diagnose airflow obstruction. Comparisons with computed tomography and morbidity indices. Ann. Am. Thorac. Soc..

[19] Melbye H, Medbø A, Crockett A (2006). The FEV1/FEV6 ratio is a good substitute for the FEV1/FVC ratio in the elderly. Prim. Care. Respir. J..

[20] Rosa FW, Perez-Padilla R (2007). Camelier a, Nascimento O a, Menezes a MB, Jardim JR. Efficacy of the FEV1/FEV6 ratio compared to the FEV1/FVC ratio for the diagnosis of airway obstruction in subjects aged 40 years or over. Braz. J. Med. Biol. Res..

[21] Vandevoorde J, Verbanck S, Schuermans D, Vincken W (2005). The role of FEV6 in the detection of airway obstruction. Respir. Med..

[22] Vandevoorde J, Verbanck S, Schuermans D, Kartounian J, Vincken W (2005). FEV1/FEV6 and FEV6 as an alternative for FEV1/FVC and FVC in the spirometric detection of airway obstruction and restriction. Chest.

[23] Vandevoorde J, Verbanck S, Schuermans D, Kartounian J, Vincken W (2006). Obstructive and restrictive spirometric patterns: fixed cut-offs for FEV1/FEV6 and FEV6. Eur. Respir. J..

[24] Thorn J, Tilling B, Lisspers K, Jörgensen L, Stenling A, Stratelis G (2012). Improved prediction of COPD in at-risk patients using lung function pre-screening in primary care : a real-life study and cost-effectiveness analysis. Prim. Care. Respir. J..

[25] van den Bemt L, Wouters BCW, Grootens J, Denis J, Poels PJ, Schermer TR (2014). Diagnostic accuracy of pre-bronchodilator FEV1/FEV6 from microspirometry to detect airflow obstruction in primary care: a randomised cross-sectional study. NPJ. Prim. Care. Respir. Med..

[26] Frith P, Crockett A, Beilby J, Marshall D, Attewell R, Ratnanesan A (2011). Simplified COPD screening: validation of the PiKo-6^®^ in primary care. Prim. Care. Respir. J..

[27] Jithoo A, Enright P, Burney P, Buist aS, Bateman ED, Tan WC (2013). Case-finding options for COPD : results from the BOLD Study. Eur. Respir. J.

[28] Gochicoa-Rangel L, Larios-Castañeda PJ, Miguel-Reyes JL, Briseño DM, Flores-Campos R, Sáenz-López JA (2014). PIKO-6 vs. forced spirometry in asthmatic children. Pediatr. Pulmonol..

[29] Kishi H, Shibata Y, Osaka D, Abe S, Inoue S, Tokairin Y (2011). FEV6 and FEV1/FEV6 in Japanese Participants of the Community-Based Annual Health Check: The Takahata Study. Intern. Med..

[30] Gleeson S, Mitchell B, Pasquarella C, Reardon E, Falsone J, Berman L (2006). Comparison of FEV6 and FVC for detection of airway obstruction in a community hospital pulmonary function laboratory. Respir. Med..

[31] Aghili R, Kia M, Meysamie A, Aghili SM, Paknejad O (2013). Fixed Cut-Off for FEV1/FEV6 and FEV6 in Detection of Obstructive and Restrictive Patterns. Iran Red Crescent Med. J..

[32] Kodgule RR, Singh V, Dhar R, Saicharan BG, Madas SJ, Gogtay Ja (2014). Reference values for peak expiratory flow in Indian adult population using a European Union scale peak flow meter. J. Postgrad. Med..

[33] Miller MR, Hankinson J, Brusasco V, Burgos F, Casaburi R, Coates a (2005). Standardisation of spirometry. Eur. Respir. J..

[34] Crapo R, Casaburi R, Coates A, Enright P, Hankinson J, Irvin C (1999). American Thoracic Society Guidelines for Methacholine and Exercise Challenge Testing—1999. Am. J. Respir. Crit. Care Med.

[35] Global Initiative for Chronic Obstructive Lung Disease (GOLD) (2014). From the global strategy for the diagnosis, management and prevention of COPD. http://www.goldcopd.org/

[36] Pellegrino R (2005). Interpretative strategies for lung function tests. Eur. Respir. J..

[37] Hajian-Tilaki K (2014). Sample size estimation in diagnostic test studies of biomedical informatics. J. Biomed. Inform..

